# Impact of Sex Differences on the Outcomes of Coronary Invasive Physiological Assessment: Long-Term Follow-Up in a Brazilian Population

**DOI:** 10.1089/whr.2023.0087

**Published:** 2024-02-12

**Authors:** Clarissa Campo Dall'Orto, Lara Eurípedes Vilela, Gilvan Vilella Pinto Filho, Marcos Raphael da Silva

**Affiliations:** Department of Hemodynamic and Interventional Cardiology, Advanced Hemodynamic Therapy Center, Brazilian Society of Health Support Hospital, Teixeira de Freitas, Bahia, Brazil.

**Keywords:** coronary disease, coronary stent, fractional flow reserve, revascularization

## Abstract

**Objective:**

This study aimed to evaluate the rate of major adverse cardiac events (MACEs; the sum of death, myocardial infarction, and revascularization rates) according to interventional strategies guided by invasive physiological methods in both sexes in a Brazilian population during long-term follow-up for an average of 2 years.

**Methods:**

This retrospective single-center study included 151 consecutive patients (232 lesions) between January 2018 and January 2022. The participants were divided into two groups: the female group (FG), comprising 59 patients with 88 lesions, and the male group (MG), comprising 92 patients with 144 lesions.

**Results:**

The FG had a greater mean age (FG: 67.96 ± 13.12 vs. MG: 62.36 ± 12.01 years, *p* = 0.009) and lower mean creatinine clearance (FG: 79.35 ± 38.63 vs. MG: 92.02 ± 38.62 mL/min, *p* = 0.02) than did the MG. The percentage of lesions in the left main coronary artery was higher in the FG than in the MG (12.5% vs. 2.78%, *p* = 0.006). The mean follow-up time was longer in the MG than in the FG (795.61 ± 350 vs. 619.19 ± 318 days, respectively; *p* = 0.001). MACE occurred in 11.86% and 13.04% of patients in the FG and MG, respectively (*p* = 0.850). Secondary outcomes, such as death, reinfarction, and the need for new revascularization, showed no significant between-sex differences.

**Conclusions:**

Our study demonstrated the safety of invasive physiological methods to determine coronary revascularization in both male and female patients in a Brazilian population, as evidenced by the low rates of adverse cardiac events and death after a long-term follow-up.

## Introduction

Functional assessment can be performed using a hyperemic method (fractional flow reserve [FFR]), whereby a hyperemia-inducing drug such as adenosine is used, as well as by nonhyperemic methods that do not require a hyperemia-inducing agent (instantaneous wave-free ratio [iFR], resting full-cycle ratio [RFR], and diastolic hyperemia-free ratio [DRF]). Numerous studies have demonstrated that compared with isolated angiography, functional assessment methods are beneficial in guiding percutaneous coronary intervention (PCI), exhibit a lower incidence of adverse cardiac events, have lower annual costs, and exhibit a performance similar to that of isolated angiography.^1–4^

Studies have investigated population characteristics such as female sex in the analysis of invasive physiological methods. Some reported differences between men and women, such as greater epicardial coronary flow^5^ and higher FFR values, with a lower revascularization rate in women when guided by FFR, demonstrating comparable clinical results regardless of sex.^6–9^ However, no study has addressed sex differences in the Brazilian population. Our primary objective was to assess whether there was a difference between sexes regarding the effectiveness of the interventional strategy guided by invasive physiological methods in a Brazilian population.

## Materials and Methods

### Study design and participants

This retrospective single-center study was conducted from January 2018 to January 2022 and included consecutive patients from a region encompassing 17 municipalities. The study was approved by the institutional ethics board of the Brazilian Society of Health Support Hospital. Written informed consent was obtained from all the patients.

### Inclusion criteria

During the study period, all consecutive patients aged ≥18 years with stable angina were included in the study if they had coronary artery stenoses ranging from 50% to 80% of the vessel diameter in at least one epicardial coronary artery and if the clinical data and angiographic appearance indicated or suggested myocardial revascularization.

### Exclusion criteria

Patients who were pregnant or exhibited hemodynamic instability at the time of the intervention (heart rate <50 beats per minute or systolic blood pressure <90 mmHg), coronary lesions with a flow <2 according to thrombolysis in the myocardial infarction scale, contraindications for PCI or drug-eluting stent (DES) implantation, severe concomitant heart valve disease, a malignant disease with poor prognosis, or life expectancy of <1 year were excluded.

### Treatment

If the invasive physiological method indicated the presence of ischemia, the patients were referred for myocardial revascularization, through either open surgery (coronay artery bypass grafting) or PCI, depending on the clinical condition at the discretion of the heart team. DES were used when PCI was performed, and patients received dual antiplatelet therapy with aspirin and a P2Y12 inhibitor for at least 6 months after the procedure.

To perform nonhyperemic invasive physiological methods such as iFR and RFR, a coronary pressure wire was advanced and equalized with the pressure sensor at the tip of the guide catheter. Next, the pressure wire was advanced into the coronary artery distal to the lesion under investigation. The iFR and RFR results were generally considered positive when the values were ≤0.89. However, some operators used a value between 0.86 and 0.93 (called a “gray area” by some authors) as an alternative to the cutoff of ≤0.89. For patients with positive iFR and RFR results, a hybrid approach with FFR was recommended (iFR/RFR followed by FFR).^8,9^ No patients underwent FFR alone without a nonhyperemic method.

In patients undergoing FFR, in addition to the previous step, maximal coronary hyperemia was induced using a central venous infusion of adenosine [140 μg/(kg·min)], and the FFR was measured. If the FFR was less than or equal to the ischemic threshold (*i.e.*, ≤0.80), revascularization was performed to address the stenosis.

All patients who underwent PCI were administered dual antiplatelet therapy comprising aspirin and a P2Y12 receptor inhibitor for at least 6 months.

### Criteria for decision making by the interventional cardiologist

**FIG. 1.**
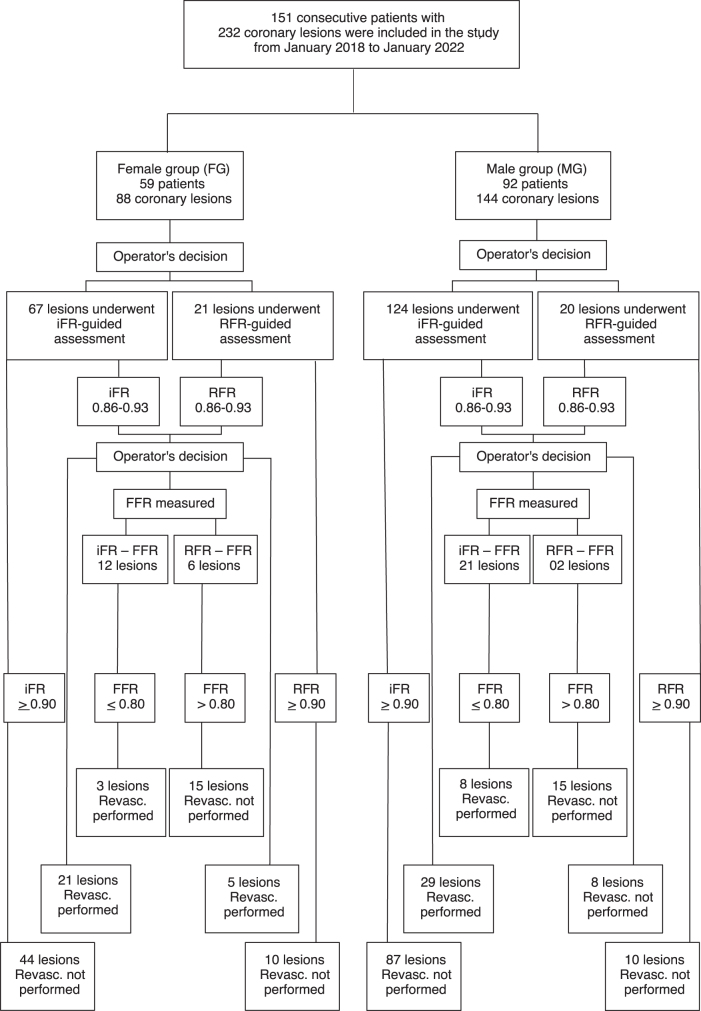
Enrollment and lesion treatment strategies. CABG, coronary artery bypass grafting; FFR, fractional flow reserve; iFR, instantaneous wave-free ratio; PCI, percutaneous coronary intervention; RFR, resting full-cycle ratio.

The interventional cardiologist followed the flowchart shown in Figure 1. Patients were divided into two groups based on sex: female group (FG) and male group (MG).

Invasive physiological assessment was performed for coronary artery lesions with stenosis ranging from 40% to 80%. If the iFR/RFR value was <0.89 or between 0.86 and 0.93, followed by an FFR <0.80, the patient was referred for revascularization and continued to receive optimal clinical treatment (referred lesions). If the iFR/RFR value was outside the specified range, the patient continued to receive optimal clinical treatment without undergoing any revascularization strategy for deferred lesions.

All revascularization procedures were performed using current standard techniques. All patients who underwent PCI received second-generation drug-eluting stents and dual antiplatelet therapy.

### iFR realization protocol

iFR was calculated by measuring the pressure gradient at rest across the coronary lesion during diastole, especially when microvascular resistance was low and stable. The measurement was performed by placing a Verrata^®^ guidewire (Philips Volcano, Rancho Cordova, CA, USA), with a pressure transducer at its distal end, through the coronary lesion.

### RFR realization protocol

The steps of the RFR realization protocol were identical to those of the iFR realization protocol. However, the guidewire (PressureWire™ X guidewire; Abbott Vascular, Santa Clara, CA, USA) used was different.

### FFR realization protocol

FFR was measured using the Verrata coronary guidewire or the PressureWire™ X guidewire, which was already positioned distal to the coronary artery lesion for iFR or RFR measurement. Adenosine was then administered to assess the pressure gradient across the lesion during maximal hyperemia. The FFR value was then calculated from the pressure ratio across the lesion during maximum hyperemia and pressure in the aorta.

Patients who underwent all three methods received intracoronary nitroglycerin and were subjected to full heparinization before the guidewire was passed through the coronary artery.

### Study objectives

The primary objective was to determine the rate of major adverse cardiac event (MACE) during the follow-up period. MACE was defined as the sum of the rates of death, MI, and the need for new revascularization. The secondary endpoints were defined as the rates of death, MI, and the need for new revascularization.

Patients were followed up through face-to-face consultation or telephone contact at 1, 3, 6, and 12 months after the procedure and periodically thereafter. To ensure minimal misclassification of results during telephone follow-up, we implement strict quality control. The following measures were implemented. (1) We provided adequate training for the professionals involved in correctly classifying the results, including providing detailed information on the classification criteria and clear guidance on how to deal with different situations. (2) We established standardized protocols for classifying results so that all professionals were able to follow the same guidelines, thus helping to minimize variability in classifications and reduce errors arising from individual interpretations.

(3) We implemented a peer review system, wherein the results for which the initial contact professionals were unsure were reviewed by another professional before being considered final; this allowed the identification of possible errors or discrepancies and guaranteed a second opinion regarding the classification. (4) To assess the accuracy of outcome classifications, regular audits were performed by professionals with the use of a reference standard by randomly selecting a sample of cases and comparing the classifications. (5) We employed feedback and continuous training of professionals based on audits and review of cases in which classification errors occurred.

(6) We monitored and analyzed data to identify trends or patterns of classification errors, thus helping to identify specific problem areas that require additional attention and the implementation of corrective measures. (7) We obtained feedback from patients through satisfaction surveys or interviews, allowing them to express their opinions and identify possible errors or problems.

The procedural success rate was defined as the percentage of cases in which the invasive physiology technique was performed successfully and provided accurate information about the physiology of the coronary arteries. Death was defined as mortality due to any cause. MI was defined *a priori* in the study protocol as the appearance of new pathological Q waves on electrocardiography in at least two contiguous leads and/or an increase in high-sensitivity troponin level by more than five times the upper limit of normal during the index hospitalization. If troponin was not administered or was not available, an increase in creatine phosphokinase-MB level by more than five times the upper limit of normal was considered.

The need for new revascularization was defined as revascularization procedures that were not included in the index procedure and were not identified during the index procedure or within the first 60 days after the procedure. Contrast-associated acute kidney injury was defined as an increase in serum creatinine of 0.5 mg/dL (44 μmol/L), or a 25% increase from baseline, within 2–5 days of the procedure.^10,11^ Bleeding was defined according to the Bleeding Academic Research Consortium criteria.^12,13^ In-hospital and follow-up MACEs were defined as the sum of the rate of MI, need for urgent (unplanned) revascularization, and death.

### Statistical analysis

Continuous quantitative variables were assessed using Student's *t*-test. Numerical values are presented as the mean ± standard deviation. Categorical variables were compared using the chi-square test or Fisher's exact test, as appropriate. A logistic regression model was used to control for the variables and assess the differences between the groups in terms of the time of the event and the outcomes. The variables were adjusted for sex, age, diabetes mellitus, unstable angina, chronic renal failure, previous MI, and significant left ventricular dysfunction.

The endpoints throughout the follow-up period were shown using Kaplan–Meier curves, and the log-rank test was used to compare the two groups.

Statistical analyses were performed using R software, version 3.3.1 (R Foundation for Statistical Computing, Vienna, Austria; https://www.R-project.org/). Statistical significance was set at *p* < 0.05.

## Results

From January 2018 to January 2022, 151 consecutive patients were recruited from a center in the state of Bahia, Brazil, and 232 coronary artery lesions were evaluated using an invasive physiological method. These patients were divided into two groups: the FG consisted of 59 female patients (88 lesions) and the MG consisted of 92 male patients (144 lesions) (Table 1).

**Table 1.** Quantification of the Sample Studied in Different Groups

Variables	FG, ***n*** (%)	MG, ***n*** (%)	***p***
Number of patients	59 (39.07)	92 (60.93)	0.02
Number of lesions	88 (37.94)	144 (62.06)	0.002
Number of lesions assessed by iFR	67 (76.14)	124 (86.11)	0.544
Number of lesions assessed by RFR	21 (23.86)	20 (13.88)	0.109
Number of lesions assessed by FFR	18 (20.45)	23 (15.97)	0.469
Number of lesions assessed by iFR+FFR	12 (13.63)	21 (14.58)	0.862
Number of lesions assessed by RFR+FFR	6 (6.81)	2 (1.38)	0.03
Number of lesions assessed by angiography/patient	1.52 ± 0.72	1.56 ± 0.76	0.747
Number of ischemic lesions/patient	0.52 ± 0.77	0.45 ± 0.67	0.516

Data are presented as numbers (percentages).

FFR, fractional flow reserve; FG, female group; iFR, instantaneous wave-free ratio; MG, male group; RFR, resting full-cycle ratio.

The demographic characteristics that were different between the two groups were as follows (Table 2): there were proportionally more men than women in our sample, and this was statistically significant (women: 39.07%, men: 60.93%; *p* = 0.02). Consequently, a greater number of assessed lesions were from men than from women; the difference was also statistically significant (lesions in women: 37.94%; lesions in men: 62.06%, *p* = 0.002). The FG exhibited a higher mean age (FG: 67.96 ± 13.12 vs. MG: 62.36 ± 12.01 years; *p* = 0.009) and lower mean creatinine clearance (FG: 79.35 ± 38.63 vs. MG: 92.02 ± 38.62 mL/min; *p* = 0.02).

**Table 2.** Baseline Clinical Characteristics of Patients

Variable	Female group (***n*** = 59), ***n*** (%)^a^	Male group (***n*** = 92), ***n*** (%)^a^	***p***
Age (years)	67.96 ± 13.12	62.36 ± 12.01	0.009
Race			
White	44 (74.58)	56 (60.87)	0.437
Black	7 (11.86)	10 (10.87)	0.866
Brown	8 (13.56)	25 (27.17)	0.108
Indigenous	0 (0)	1 (1.09)	0.424
Average body mass index^b^	26.29 ± 5.14	26.42 ± 3.82	0.865
Body mass index^b^ <18.5	2 (3.39)	2 (2.17)	0.658
Body mass index^b^ ≥30	10 (16.95)	13 (14.13)	0.687
Creatinine clearance ratio^c^ (mL/min)	79.35 ± 38.62	92.02 ± 42.20	0.02
Creatinine clearance^c^ ≥90 mL/min	19 (32.2)	49 (53.26)	0.111
Creatinine clearance^c^ 60–89 mL/min	20 (33.9)	21 (22.83)	0.262
Creatinine clearance^c^ 30–59 mL/min	16 (27.12)	19 (20.65)	0.47
Creatinine clearance^c^ 15–29 mL/min	2 (3.39)	1 (1.09)	0.333
Creatinine clearance^c^ ≤15 mL/min	1 (3.39)	1 (2.17)	0.753
Hypertension	49 (83.05)	81 (88.04)	0.812
Dyslipidemia	42 (71.19)	68 (73.91)	0.884
Family history of CAD	32 (54.24)	38 (41.3)	0.35
Current smoker	6 (10.17)	11 (11.96)	0.761
Diabetes mellitus	24 (40.68)	31 (33.7)	0.554
Previous MI	13 (22.03)	29 (0)	0.335
Previous PCI	22 (37.29)	42 (45.65)	0.515
Previous CABG	5 (8.47)	4 (4.35)	0.326
Previous stroke	1 (1.69)	2 (2.17)	0.84
Peripheral vascular disease	5 (8.47)	4 (4.35)	0.326
COPD	1 (1.69)	3 (3.26)	0.568

Data are presented as the mean ± standard deviation or number (percentage). There were significant differences in baseline characteristics between the two groups regarding age and mean creatinine clearance values.

^a^ Number of patients.

^b^ Body mass index is the weight in kilograms divided by the square of the height in meters.

^c^ Creatinine clearance expressed in mL/min was estimated using the Cockroft and Gault formula.

CABG, coronary artery bypass grafting; CAD, coronary artery disease; COPD, chronic obstructive pulmonary disease; MI, myocardial infarction; PCI, percutaneous coronary intervention.

The technical characteristics are presented in Table 3, and procedural characteristics related to the variables based on the number of lesions are summarized in Table 4. The severity of coronary artery disease (CAD) was similar between women and men, except for the higher percentage of lesions assessed in the left main coronary artery in the FG than that in the MG (12.5% vs. 2.78, respectively; *p* = 0.006). RFR was performed more often in the FG than in the MG (6% vs. 2%, respectively; *p* = 0.03). The mean iFR value tended to be lower in the FG than in the MG (mean value: 0.89 ± 0.09 vs. 0.92 ± 0.07, respectively; *p* = 0.05). The concordance rate between the nonhyperemic methods and FFR did not present a statistically significant difference between the groups.

**Table 3.** Procedural Characteristics Related to Variables by the Number of Patients

Variables	FG (***n*** = 59), ***n*** (%)^a^	MG (***n*** = 92), ***n*** (%)^a^	***p***
Radial artery approach	48 (81.36)	81 (88.04)	0.749
Volume of ionic contrast medium (mL)	146.52 ± 58.75	164.59 ± 64.50	0.09
Procedure time (minutes)	56.30 ± 30.26	55.47 ± 32.89	0.182
Number of affected arteries^b^			
Single vessel	23 (38.98)	24 (26.09)	0.23
Two vessels	16 (27.12)	35 (38.04)	0.325
Three vessels	20 (33.9)	33 (35.87)	0.863
Left ventricular ejection fraction			
≥55%	34 (57.63)	47 (51.09)	0.667
≥40% and <55%	6 (10.17)	12 (13.04)	0.636
≥30% and <40%	6 (10.17)	19 (20.65)	0.148
<30%	1 (1.69)	3 (3.26)	0.568
Left ventriculography not performed	12 (20.34)	11 (11.96)	0.233

Data are presented as mean ± standard deviation or number (percentage).

^a^ Number of patients.

^b^ Presence of >50% stenosis in the epicardial coronary artery.

**Table 4.** Procedural Characteristics Related to Variables by Number of Lesions

	FG (***n*** = 88), ***n*** (%)^a^	MG (***n*** = 144), ***n*** (%)^a^	***p***
Lesion territory			
Right coronary artery and branches	12 (13.64)	21 (14.58)	0.862
Left circumflex artery and branches	13 (14.78)	25 (17.36)	0.66
Left anterior descending artery and branches	50 (56.81)	90 (62.5)	0.668
Intermediate	2 (2.27)	4 (2.78)	0.818
Left main	11 (12.5)	4 (2.78)	0.006
AHA classification			
A	5 (5.68)	9 (6.25)	0.868
B1	34 (38.64)	58 (40.28)	0.87
B2	21 (23.86)	24 (16.67)	0.272
C	28 (31.82)	53 (36.81)	0.589
SCAI classification			
I	61 (69.32)	91 (63.19)	0.665
II	27 (30.68)	53 (36.81)	0.503
Calcification			
Moderate	15 (17.05)	18 (12.5)	0.406
Severe	4 (4.55)	1 (0.69)	0.05
Intracoronary adenosine	18 (20.45)	23 (15.97)	0.469
iFR performed^b^	67 (76.14)	124 (86.11)	0.544
RFR performed^b^	21 (23.86)	18 (12.5)	0.308
FFR performed^b^	18 (20.45)	23 (15.97)	0.469
Revascularized lesions	29 (32.95)	45 (31.25)	0.846
ICP of functionally significant lesions^c^	27 (30.68)	42 (29.17)	0.857
CABG of functionally significant lesions^c^	2 (2.27)	3 (2.08)	0.924
Changed the interventional cardiologist initial decision	23 (26.14)	38 (26.39)	0.974
Concordant results for hyperemic and nonhyperemic methods			
Total nonhyperemic methods followed by FFR	18 (20.45)	23 (15.97)	0.465
iFR followed by FFR:	12 (13.63)	21 (14.5)	0.62
iFR + followed by FFR −	3 (3.4)	4 (2.77)	0.791
iFR + followed by FFR +	2 (2.27)	3 (2.08)	0.924
iFR − followed by FFR −	8 (9.09)	10 (6.94)	0.584
iFR − followed by FFR +	0 (0)	4 (2.77)	0.119
RFR followed by FFR:	6 (6.81)	2 (1.38)	0.03
RFR − followed by FFR −	6 (6.81)	2 (1.38)	0.03
Lesion with QCA <70%	55 (62.5)	92 (63.8)	0.919
Lesions with QCA <70% and positive physiological method:	15 (17.04)	18 (12.5)	0.406
iFR positive	9 (10.22)	12 (8.33)	0.656
RFR positive	5 (5.68)	3 (2.08)	0.16
FFR positive	1 (1.13)	3 (2.08)	0.596
Lesion with QCA ≥70%	33 (37.5)	52 (36.11)	0.884
Lesions with QCA ≥70% and positive physiological method:	22 (25)	33 (22.91)	0.776
iFR positive	17 (19.31)	25 (17.36)	0.754
RFR positive	4 (4.54)	4 (2.77)	0.49
FFR positive	1 (1.13)	4 (2.77)	0.412
Diameter stenosis, mean (SD)	61.02 ± 13.73	58.81 ± 12.08	0.216
Average iFR, mean (SD)	0.89 ± 0.09	0.92 ± 0.07	0.05
Average RFR, mean (SD)	0.89 ± 0.09	0.90 ± 0.04	0.637
Average FFR, mean (SD)	0.86 ± 0.06	0.84 ± 0.04	0.153
No. of lesions assessed/patient	1.52 ± 0.72	1.56 ± 0.76	0.747
No. of ischemic lesions/patient	0.52 ± 0.77	0.45 ± 0.67	0.516

Data are presented as the mean ± standard deviation or number (percentage).

^a^ Number of lesions evaluated.

^b^ Type of physiological assessment of vessels.

^c^ Functionally significant lesions with an iFR/RFR ≤0.89 or FFR <0.80. iFR values between 0.86 and 0.93 resulted in conversion of the procedure to FFR at the interventional cardiologist discretion.

AHA, American Heart Association; SCAI, Society for Cardiovascular Angiography and Interventions; QCA, quantitative coronary angiography.

Similarly, there was no statistically significant difference in the percentage of lesions evaluated using quantitative coronary angiography (QCA) categorized as <70% or ≥70% compared with the results obtained from the nonhyperemic methods. There was no difference between the number of assessed lesions per patient and number of ischemic lesions per patient. There was also no difference in the rate of revascularization based on the methods used in the two sexes.

The in-hospital evolution is given in Table 5, with no difference between the two groups with regard to the rate of renal failure, bleeding, new MI, need for new (unplanned) emergency revascularization, and death.

**Table 5.** In-Hospital Evolution

	FG (***n*** = 59), ***n*** (%)	MG (***n*** = 92), ***n*** (%)	***p***
Procedure success^a^	57 (96.61)	90 (97.8)	0.958
MI	1 (1.69)	0 (0)	0.214
Contrast-associated AKI^b^	2 (3.38)	4 (4.34)	0.777
Bleeding^c^	1 (1.69)	2 (2.17)	0.84
Stroke	0 (0)	0 (0)	—
Coronary dissection	1 (1.69)	0 (0)	0.214
Need for urgent (unplanned) revascularization	1 (1.69)	0 (0)	0.214
Puncture site infection	0 (0)	0 (0)	—
In-hospital MACE^d^	2 (3.38)	0 (0)	0.08

^a^ The procedural success rate was defined as the percentage of cases in which the technique was performed successfully and provided accurate information about the physiology of the coronary arteries.

^b^ Contrast-associated AKI: Defined as an increase in serum creatinine of 0.5 mg/dL (44 μmol/L), or a 25% increase from baseline, within 2–5 days of the procedure.^1,2^

^c^ Used the criteria of the BARC consortium.^3,4^

^d^ In-hospital MACE, defined as the sum of the MI rate, need for urgent (unplanned) revascularization and death.

AKI, acute kidney injury; MACE, major adverse cardiac events.

The variables were adjusted for sex, age, diabetes mellitus, unstable angina, chronic renal failure, previous MI, and significant left ventricular dysfunction using the multivariate logistic regression model, and none was found to be potentially confounding variables. The mean follow-up time was longer in the MG than in the FG (619.19 ± 318 vs. 795.61 ± 350 days, respectively; *p* = 0.001). The rates of loss to follow-up were 8.4% and 7.6% in the FG and MG, respectively (*p* = 0.859; Table 6).

**Table 6.** Long-Term Follow-Up

Patients followed	FG (***n*** = 59), ***n*** (%)^a^	MG (***n*** = 92), ***n*** (%)^a^	***p***
Average segment time [mean (SD)]	619.19 ± 318	795.61 ± 350	0.001
Lost to follow-up	5 (8.4)	7 (7.6)	0.859
Repeat coronary angiography nonurgent	7 (11.86)	16 (17.39)	0.426
Repeat revascularization nonurgent	7 (11.86)	6 (6.52)	0.297
TVR nonurgent	6 (10.16)	6 (6.52)	0.456
TLR nonurgent	5 (8.47)	5 (5.43)	0.494
Nonfatal MI	1 (1.69)	2 (2.17)	0.84
Death from all causes	2 (3.38)	4 (4.34)	0.777
MACE	7 (11.86)	12 (13.04)	0.85

Data are presented as mean ± standard deviation (SD) or number (percentage).

^a^ Number of patients.

TLR, target lesion revascularization; TVR, target vessel revascularization; SD, standard deviation.

**FIG. 2.**
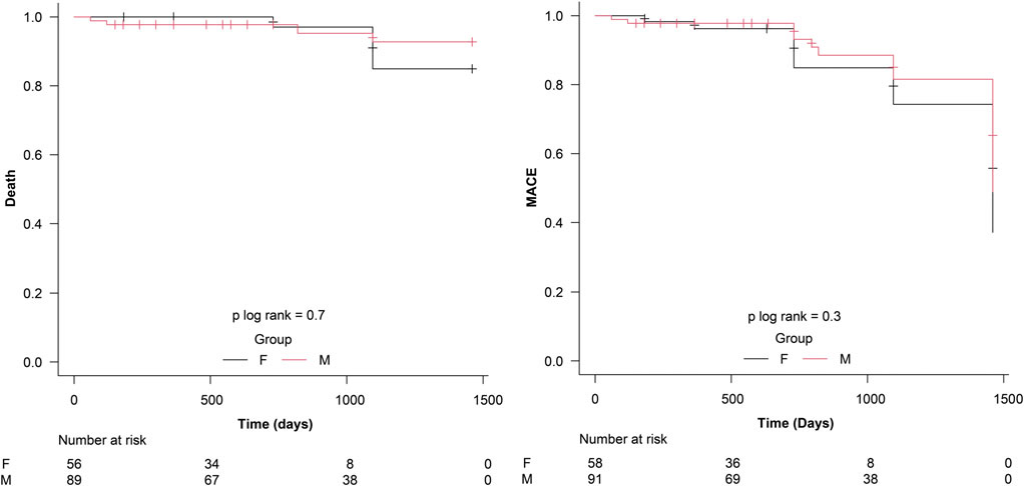
Kaplan–Meier curves for death-free survival and MACE in the follow-up period for an average of 2 years. **(A)** MACE-free survival. **(B)** Survival free of all-cause death. F, female group; M, male group; MACEs, major adverse cardiac events.

During the follow-up period (mean: 2 years), the primary outcomes were 11.86% and 13.04% in the FG and MG, respectively (*p* = 0.850). Regarding secondary outcomes, there was no significant difference in the rates of death from all causes (FG: 3.38% vs. MG: 4.34%, *p* = 0.777), reinfarction (FG: 1.69% vs. MG: 2.17%, *p* = 0.84), and the need for new revascularization (FG: 11.86% vs. MG: 6.52, *p* = 0.297) between the two groups. The survival analysis with the Kaplan–Meier curve and log-rank p of the death rate and MACE in both groups are shown in Figure 2.

## Discussion

To our knowledge, this study is the first to investigate sex differences regarding the nuances of invasive physiological assessment in a Brazilian population. Moreover, among the studies that evaluated this issue, our study presents one of the longest follow-up times in the global literature, with an average of 2 years of follow-up.

In our sample, which comprised consecutive patients referred to the hemodynamics laboratory by the assistant cardiologist, the number of men was significantly higher than that of women, reflecting the well-known epidemiology of CAD in Brazil and the world. Significant differences exist between women and men in the epidemiology, diagnosis, and prognosis of CAD. Moreover, women are often under-represented in randomized cardiovascular trials, and data suggest that in clinical practice, women are not referred for appropriate therapeutic procedures as often as men are.^14–16^

There are sex differences in PCI, whether guided by angiography alone or by functional assessment. The severity of CAD is overestimated in women compared with that in men on visual analysis, even when using measurements performed by QCA software.^17^ Women with CAD are less likely to be revascularized than are men based on angiography alone. Some studies have demonstrated that this difference persists even after functional evaluation using FFR.^18^

We observed higher FFR values in women than in men, although the difference was not statistically significant. In our study cohort, there was no evidence of a difference in the rate of lesions assessed or in the rate of ischemic lesions between the sexes. Several studies reported significantly lower FFR values in male patients than in female patients.^9,19,20^ In the Fractional Flow Reserve versus Angiography for Guiding Percutaneous Coronary Intervention substudy, female patients had higher FFR values for a given severity of coronary stenosis than did male patients, and the proportion of functionally significant lesions was lower in women than in men.^9^

In our study, we also observed a high prevalence of iFR^+^/FFR^−^ in women and of iFR^−^/FFR^+^ in men, but this did not reach statistical significance. A study by Aoi et al., which evaluated the concordance rate between iFR and FFR, concluded that the iFR^+^/FFR^−^ discordance was greater in women than in men and that the iFR^−^/FFR^+^ discordance was greater in men than in women.^21^ Yonetsu et al. reported that discordant results with iFR^−^/FFR^+^ were observed more frequently in men than in women.^22^

Our findings indicate that the strategy for assessing coronary physiology is safe and effective in both male and female patients in a Brazilian population, with a mean follow-up of 700 days. In both groups, patients exhibited low rates of adverse cardiac events and death after a long-term follow-up, despite the heterogeneity of the groups, which reflects a real-world population. The results of our series align with other daily clinical practice records that assessed the benefits of coronary revascularization guided by invasive physiological methods in both sexes.^6,9,19^

Observational data, such as those obtained from clinical records, are important because they complement the scientific evidence from randomized controlled trials and demonstrate effectiveness in clinical practice.

Our study's main limitations are that it was a single-center registry-based study and not a randomized trial. In addition, the number of patients was relatively small, and some patients were lost to follow-up. The groups were not completely homogeneous. The sample comprised mainly men, with a higher percentage of older patients and patients with lower creatinine clearance in the FG and a greater presence of left main lesions in women. But despite these demographic and angiographic differences between the groups, there was no difference in the incidence of primary and secondary outcomes.

In addition, the lack of absolute standardization of the interventional cardiologist protocols was a limitation. The interventional cardiologist decided to perform FFR following the nonhyperemic method if values were in the range of 0.86–0.93 or <90. Furthermore, all indices were not measured for all patients, so a direct comparison between the techniques in both sexes could not be made. We also did not assess microvascular function with coronary flow reserve or index of myocardial resistance because the required equipment was not commercially available in Brazil during the study period.

## Conclusions

Our study showed the safety of the invasive physiological method as an indicator of coronary revascularization in daily clinical practice in both male and female patients in a real-world setting in a Brazilian population, as evidenced by the low rates of adverse cardiac events and death after a long-term follow-up.

## Abbreviations Used

AHA: American Heart Association
AKI: acute kidney injury
CABG: coronary artery bypass grafting
CAD: coronary artery disease
COPD: chronic obstructive pulmonary disease
FFR: fractional flow reserve
FG: female group
iFR: instantaneous wave-free ratio
MACE: major adverse cardiac event
MG: male group
MI: myocardial infarction
PCI: percutaneous coronary intervention
QCA: quantitative coronary angiography
RFR: resting full-cycle ratio
SCAI: Society for Cardiovascular Angiography and Interventions
SD: standard deviation.
TLR: target lesion revascularization
TVR: target vessel revascularization
